# Comparison of immune responses to *Loa loa* stage-specific antigen extracts in *Loa loa*-exposed BALB/c mice upon clearance of infection

**DOI:** 10.1186/s13071-020-3921-x

**Published:** 2020-02-07

**Authors:** Valerine C. Chunda, Manuel Ritter, Ayukenchengamba Bate, Narcisse V. T. Gandjui, Mathias E. Esum, Fanny F. Fombad, Abdel J. Njouendou, Patrick W. C. Ndongmo, Mark J. Taylor, Achim Hoerauf, Laura E. Layland, Joseph D. Turner, Samuel Wanji

**Affiliations:** 1 Parasite and Vector Biology Research Unit, Department of Microbiology and Parasitology, Faculty of Science, University of Buea, P.O. Box 63, Buea, Cameroon; 2 Research Foundation in Tropical Diseases and the Environment, P.O. Box 474, Buea, Cameroon; 3 Institute of Medical Microbiology, Immunology and Parasitology (IMMIP), Medical Faculty, University of Bonn, Bonn, Germany; 4 Centre for Drugs and Diagnostics Research, Department of Tropical Disease Biology, Liverpool School of Tropical Medicine, Pembroke Place, Liverpool, L3 5QA UK; 5 German Centre for Infection Research (DZIF), Bonn-Cologne partner site, Bonn, Germany

**Keywords:** Recall immune responses, Re-stimulation, Immunoglobulins, Chemokines, Cytokines, Microfilariae, Adult worms, Larvae, *Loa loa* antigen extract

## Abstract

**Background:**

Different immune mechanisms are capable of killing developmental stages of filarial nematodes and these mechanisms are also likely to vary between the primary and a challenge infection. However, the lack of a detailed analysis of cytokine, chemokine and immunoglobulin levels in human loiasis is still evident. Therefore, detailed analysis of immune responses induced by the different developmental stages of *Loa loa* in immune-competent BALB/c mice will aid in the characterization of distinct immune responses that are important for the immunity against loiasis.

**Methods:**

Different developmental stages of *L. loa* were obtained from human peripheral blood (microfilariae, MF), the transmitting vector, *Chrysops* (larval stage 3, L3) and infected immune-deficient BALB/cRAG2γc^−/−^ mice (L4, L5, adult worms). Groups of wildtype BALB/c mice were then injected with the isolated stages and after 42 days post-infection (pi), systemic cytokine, chemokine and immunoglobulin levels were determined. These were then compared to *L. loa*-specific responses from *in vitro* re-stimulated splenocytes from individual mice. All parameters were determined using Luminex technology.

**Results:**

In a pilot study, BALB/c mice cleared the different life stages of *L. loa* within 42 days pi and systemic cytokine, chemokine and immunoglobulin levels were equal between infected and naive mice. Nevertheless, *L. loa*-specific re-stimulation of splenocytes from mice infected with L5, MF or adult worms led to induction of Th2, Th17 and chemokine secretion patterns.

**Conclusions:**

This study shows that although host immunity remains comparable to naive mice, clearance of *L. loa* life-cycle development stages can induce immune cell memory leading to cytokine, chemokine and immunoglobulins secretion patterns which might contribute to immunity and protection against reinfection.
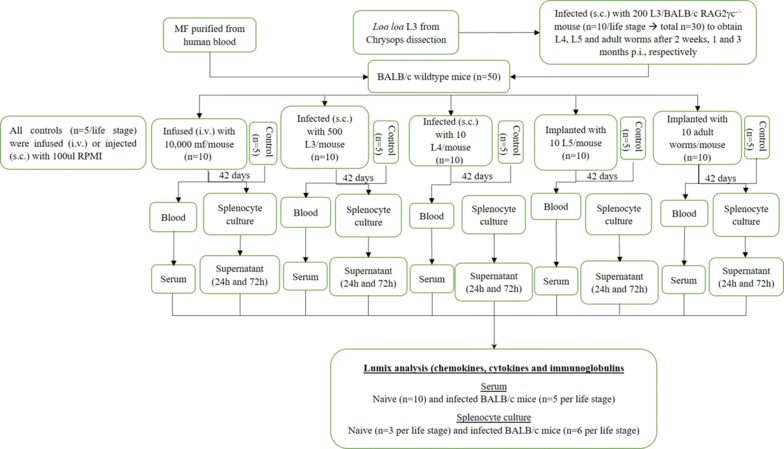

## Background

Loiasis is caused by the filarial nematode *Loa loa* that is endemic in equatorial rainforest regions of Central and West Africa. As with other filariae, humans are infected through a bite of an insect vector; here flies of the genus *Chrysop*s which are transmitting infective third-stage larvae (L3) into the bite wound. L3 then migrate through subcutaneous tissues were they mature to adult worms that produce microfilariae (MF) which can be found in peripheral blood and other body fluids [1, 2]. Since adult worms migrate under the skin and the conjunctiva, *L. loa* is often referred to African eye worm [1]. Although most infections remain asymptomatic, clinical symptoms can occur and are characterized by oedemas, Calabar swellings, purities and arthralgia [1, 3]. Moreover, high MF loads (> 30,000 MF/ml) are associated with severe encephalitis in individuals who were treated with ivermectin or diethylcarbamazine, drugs against onchocerciasis [4–7], another filarial disease. Thus, knowledge about the distribution of *L. loa* in regions were the African Programme for Onchocerciasis Control (APOC) is running is essential [8] and immune responses induced by the infection need to be taken into consideration to improve management of filarial-related adverse responses in man.

Interestingly, only a few human studies [9–12] or experiments with *L. loa*-infected mandrills or rhesus monkeys [13, 14], have analysed immunological responses during *L. loa* infection. In regard to immunoglobulins, it has been shown that IgG and IgE subclasses, especially non-specific polyclonal IgE and elevated levels of antigen-specific IgG4 are associated with *L. loa* infection [12, 13, 15, 16]. However, since all other immunoglobulin isotypes were shown to be important during other filarial infections [17–19], their role during loiasis remains uncertain. Knowledge of cytokine and chemokine responses based on a few studies using human peripheral blood mononuclear cells (PBMC), co-infection studies and experimental infections of mandrills or rhesus monkeys which suggest that Th1 and Th2 immune responses [9, 14] and eosinophil-associated cytokines [10] play a major role during *L. loa* infections. Nevertheless, a comprehensive analysis of cytokine, chemokine and immunoglobulin responses elicited throughout infection and the potential immunomodulatory capacity and mechanisms of *L. loa* remain unknown.

To decipher *L. loa*-specific immune responses in more detail, we performed an initial set of experiments that compared systemic and recall responses in groups of immune-competent BALB/c mice that had been exposed to different life stages of the filariae (Additional file 1: Figure S1). Whereas systemic cytokine, chemokine and immunoglobulin levels were comparable between *L. loa*-exposed and naive mice on 42 days post-infection (pi) (a time point where infection is cleared), we observed enhanced immunoglobulin, cytokine and chemokine levels in splenocytes from *L. loa*-exposed BALB/c mice upon re-stimulated with *L. loa* stage-specific antigen extracts. In summary, these findings show that although systemic host immunity was comparable to unexposed animals, developmental stages of *L. loa* trigger immune cells during an ongoing filarial infection leading to induction of recall immune responses that might be important for immunity against reinfections.

## Methods

### Collection, maintenance and dissection of *Chrysops* flies

*Chrysops* flies were captured in the Bakundu reserve forest in Meme Division (9°25′ E, 4°30′ N) in the South-West region of Cameroon. Flies were fed on a consenting *L. loa* positive individual with a microfilaria load above 10,000 MF/ml of blood. After a blood meal, flies were collected *via* aspiration in a dark net and then kept in separate 50-ml tubes which were filled a quarter with Plaster of Paris that formed a cement layer and helped to retain moisture. Engorged *Chrysops* were fed daily with 15% sucrose solution and kept at 23 °C for 2 weeks, the time required for the development of L3. After 14 days, flies were washed with distilled water containing 4% Tween20 (Sigma-Aldrich, St. Louis, USA) and then rinsed with distilled water in Petri dishes (Falcon, New York, USA). L3 were then isolated from the *Chrysops* in dissecting medium containing RPMI-1640 medium (Sigma-Aldrich) supplemented with a 2% antibiotic cocktail (pencillin-streptomycin-neomycin; Thermo Fisher Scientific, Schwerte, Germany) using a dissecting microscope (Motic, Wetzlar, Germany). The head, thorax and abdomen were separated and dissected in individual Petri dishes containing dissecting medium. L3 were allowed to migrate out of the various parts and washed 4 times in dissecting medium. L3 were then counted, pooled and either used for infection of mice or frozen at − 80 °C for antigen preparation.

### Isolation of *Loa loa* microfilariae from human peripheral blood

Whole blood samples (4 ml) were collected from a donor infected with *L. loa* and MF were obtained using version of a previously described protocol [20, 21]. In brief, 2 ml of whole blood was layered onto 2 ml-modified Percoll gradient (Sigma-Aldrich) in a 15-ml tube (Falcon) and centrifuged at 2000× *rpm* for 20 min without brake using a bench-top centrifuge (Human Diagnostics, Wiesbaden, Germany). Using a Whatman^®^ filter paper (pore size 5 µm) (Merck Millipore, Tullagreen, Ireland) placed in a filter paper holder, a dropper was used to discard the topmost part containing the serum. Then, the filter was mounted on another 15-ml tube and the whitish area containing the parasite was then filtered using a syringe (Terumo, Tokyo, Japan). The filter paper was then removed with a sterile pipette and placed in a Petri dish (Falcon) containing RPMI medium (Sigma-Aldrich) to aid migration of MF out of the paper into the medium. MF number and motility was determined using a dissecting microscope Leica M80 (Leica, Singapore, Republic of Singapore). MF were either frozen at − 80 °C for antigen preparation or used for infection of wildtype BALB/c mice.

### Isolation of host-derived *Loa loa* worm stages from infected mice

Female and male BALB/c RAG2γc^−/−^ mice were subcutaneously infected with 100 L3 in 100 µl RPMI-1640 (Sigma-Aldrich) that were isolated from *Chrysops* flies. To obtain L4, L5 and adult worms, mice were sacrificed and dissected 15 days, 1 and 3 months pi, respectively. To obtain parasite life stages, several organs (subcutaneous tissue, muscle tissue, peritoneal cavity, liver, lungs and heart) were placed into a Petri dish (Falcon) containing RPMI-1640 (Sigma-Aldrich) and numbers and motility was determined using a dissecting microscope Leica M80 (Leica). Obtained parasites were either frozen at − 80 °C for antigen preparation or used for infection of wildtype BALB/c mice.

### Pre-clinical experimental studies with different *Loa loa* life-stages

Simultaneously, groups of female and male wildtype BALB/c mice were exposed to either: (i) a s.c. injection of 500 L3 in 100 µl RPMI-1640 medium isolated from *Chrysops* flies; (ii) 10,000 MF *via* the tail vein in 100 µl RPMI-1640 medium isolated from human peripheral blood [22], or (iii) 10 L4; (iv) 10 L5; and (v) 10 adult worms all isolated from infected-BALB/cRAG2γc^−/−^ mice. Whereas L4 were injected s.c. in 100 µl of RPMI-medium, L5 and adult stages were implanted as previously described [23]. In short, mice were anesthetized with ketamine (Ketaset, 70 mg/kg; Zoetis, Parsippany-Troy Hills Township, New Jersey, USA) and medetomidine (Domitor, 0.8 mg/kg; Zoetis), flanks were shaved, dosed with betadine and following a small incision, L5 or adult worms were implanted into the mice. The incised area was then sutured and a s.c. shot of penicillin (12.06 mg/ml) was administered based on mouse weight (i.e. 100 µl/20 g) at the back of the neck followed by 200 µl antiserdan to wake the mouse up.

### Analysis of *Loa loa-*exposed wildtype BALB/c mice

Six weeks (42 days) pi, mice were sacrificed by exposing them to increased carbon dioxide (CO_2_) concentration. Then, the thoracic regions were opened, and cardiac blood was collected using a 1-ml insulin syringe (Terumo, Leuven, Belgium). Subsequently, blood was centrifuged at 2500× *rpm* for 10 min using a PRISMR centrifuge (Labnet, New Jersey, USA) and serum was collected and kept at − 80 °C for Luminex-based analysis of immunoglobulins, cytokines and chemokines. Additionally, spleens were isolated from individual mice for stage-specific re-stimulation assays.

### Preparation of parasite antigen extract

Adult worms, L3, L4 and L5 were thawed and concentrated by centrifuging at 1500× *rpm* for 5 min using a bench-top centrifuge (Human Biochemica und Diagnostica GmbH, Wiesbaden, Germany). Parasites were then mechanically minced on ice in cold sterile endotoxin-free PBS (Sigma-Aldrich). Insoluble material was removed by centrifugation at 2000× *rpm* for 10 min at 4 °C. The resulting soluble parasite antigen extract was then filtered through a 0.22-µm pore size filter (Merck Millipore) and protein concentration was determined by a Bradford assay (Cytoskeleton, Denver, USA) according to manufacturer’s description. Aliquots were frozen at − 80 °C until required.

### Re-stimulation of splenocytes

Isolated spleens from individual mice were crushed through a sieve (Corning, Durham, USA) and red blood cells were lysed for 5 min in the dark using lysing buffer (Sigma-Aldrich). Cell suspensions were then centrifuged for 5 min at 2000× *rpm* using a PRISMR centrifuge (Labnet) and cell numbers in the resulting pellet were counted using a cell counting chamber (VWR, Pennsylvania, USA). 1 × 10^6^ splenocytes were plated/well into 48-well culture plates (Costar, Kennebunk, USA) and left either un-stimulated or re-stimulated with 100 µg/ml of stage-specific parasite extract in a total of 800 µl culture medium (RPMI-1640 containing 10% FCS and 0.4% beta-mecaptoethanol; Sigma-Aldrich) at 37 °C and 5% CO_2_ for 48 h (cytokines) and 72 h (immunoglobulins). Thereafter, culture supernatants were removed and frozen at − 20 °C until cytokine/chemokine/immunoglobulin levels were determined by Luminex technology.

### Analysis of immunoglobulins, cytokines and chemokines levels using Luminex technology

Systemic immunoglobulin, cytokine and chemokine levels from individual mouse serum samples and supernatants of the splenocyte re-stimulation assays were determined using a ProcartaPlex Mouse Antibody Isotyping Panel 7 plex and a ProcartaPlex Mouse Cytokine/Chemokine Panel 1 26 plex (eBioscience, Frankfurt, Germany) according to the manufacturer’s instructions. The limits (upper limit of quantification/lower limit of quantification) of the cytokines and chemokines in pg/ml were as follows: eotaxin (2400/0.59), GM-CSF (10,400/2.54), CXCL1 (6400/1.56), IFN-γ (4250/1.04), TNF-α (12,800/3.13), IL-10 (9300/2.27), IL-12p70 (3900/3.81), IL-13 (11,000/2.69), IL-17A (5050/1.23), IL-18 (38,750/38), IL-1β (4800/1.17), IL-2 (6050/1.48), IL-22 (46,900/11), IL-23 (52,100/13), IL-27 (11,000/2.69), IL-4 (5350/1.31), IL-5 (9950/2.43), IL-6 (21,500/5.25), IL-9 (66,700/16), CXCL10 (1750/0.43), MCP-1 (31,200/7.62), MCP-3 (1300/1.27), MIP-1α (2000/0.49), MIP-1β (1338/1.31), MIP-2 (3100/3.03) and RANTES (3075/3.00). Results from the analysis were acquired using a MAGPIX Luminex system (Luminex Cooperation, Austin, USA) and analyzed with ProcartaPlex Analyst software 1.0 (eBioscience). Levels of immunoglobulins, cytokines and chemokines from the splenocytes which were re-stimulated with the different parasite antigen extracts were normalized by subtracting background levels of un-stimulated splenocytes. An overview of the study design is shown in Additional file 1: Figure S1.

### Statistical analysis

Statistical analyses were performed using the software SPSS 22 (IBM, Armonk, NY, USA) and the PRISM 7 programme (GraphPad Software Inc., La Jolla, USA). Variables did not meet assumption to allow parametric analysis, therefore to compare more than two groups, a Kruskal-Wallis test (systemic immune responses) or Friedman test (cytokine, chemokine and immunoglobulin levels from re-stimulated splenocytes) was performed and, if significant, followed by a Dunn’s *post-hoc* multiple comparison test for a further comparison of the groups.

## Results

### Comparable immunoglobulin levels in BALB/c upon parasite clearance

There is a growing body of evidence indicating that chronic helminth infections may influence the onset and outcome of further illnesses and disease such as allergies and tuberculosis. Due to the moderate pathologies associated with *L. loa* and *Mansonella perstans* infections, there have been few studies of the immune responses provoked by these filariae. To gain an impression as to whether *L. loa* imprint a long-lasting immune signature following clearance of infection, which might determine how the host responds to unrelated diseases at a later date, we designed an initial study using immunocompetent BALB/c mice (Additional file 1: Figure S1). In short, we exposed groups of mice to either MF, L3, L4, L5 or adult worms. Levels of immunoglobulins, cytokines and chemokines in the serum of individual mice were measured on day 42 pi. At this time point, no parasitic stages were observed in any group which supports our earlier *in vivo* studies using wildtype and cytokine-deficient BALB/c strains [24, 34]. Thus, we considered that sufficient time had passed so that retained infection profiles and memory responses could be evaluated. Interestingly, systemic total immunoglobulin patterns in the serum were not significantly regulated between naive mice and those exposed to the different *L. loa* life stages using a Kruskal-Wallis test followed by Dunn’s *post-hoc* multiple comparison test (Additional file 2: Figure S2). Nevertheless, exposure to adult stages appeared to reduce IgG1, IgG2b and IgM when compared to naive groups, albeit not significantly.

Levels of pro-inflammatory and Th1 cytokines (Additional file 3: Figure S3) as well as Th2 and Th17 cytokines (Additional file 4: Figure S4) were also comparable between naive and *L. loa*-exposed mice. Moreover, 10 chemokine parameters (Additional file 5: Figure S5) were also comparable between the groups using a Kruskal-Wallis test followed by Dunn’s *post-hoc* multiple comparison test. Albeit non-significant, some changes were seen between naive basal levels and adult stages, elevated IL-27 and IL-9 (Additional file 3: Figure S3h and Additional file 4: Figure S4d) and reduced CXCL-10, MIP-1β (Additional file 5: Figure S5b, g). Regarding L5, elevated IL-23, IL-6 and CXCL-1 (Additional file 3: Figure S3g, Additional file 4: Figure S4c, Additional file 5: Figure S5a) with reduced CXCL-10, MIP-1a, MIP-1b and RANTES (Additional file 5: Figure S5b, f, g, j) were detected.

### Enhanced IgG2a, IgE and IgM secretion upon stage-specific re-stimulation with MF antigen extract

**Fig. 1:**
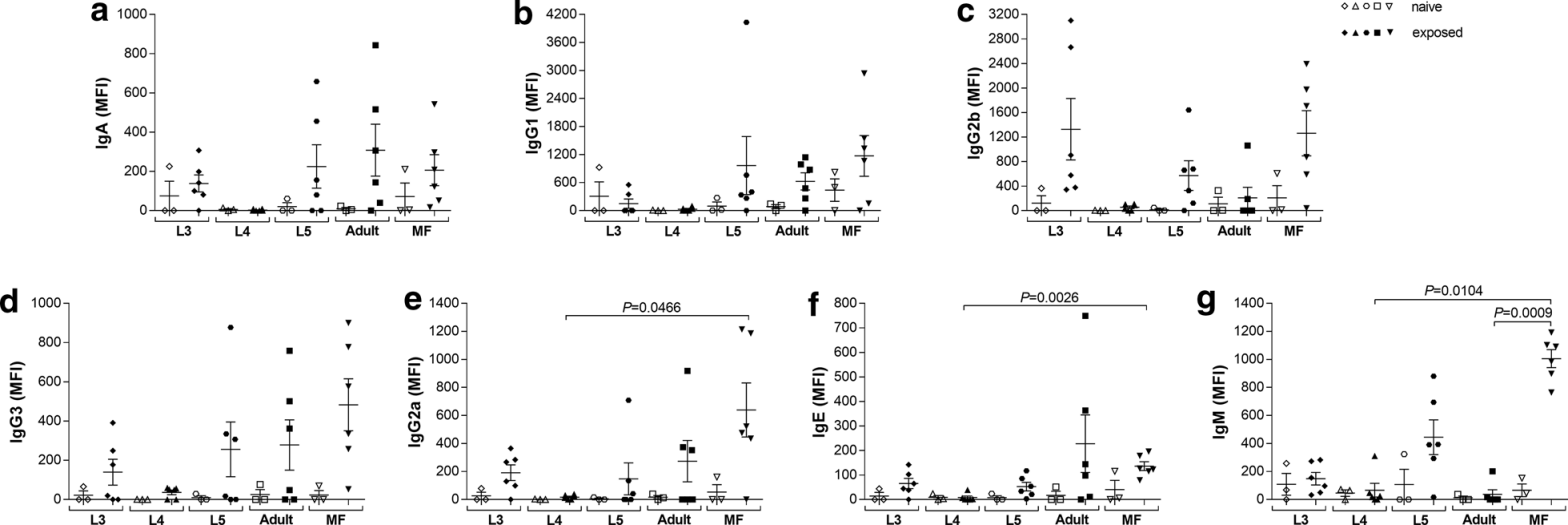
MF re-stimulated splenocytes from MF-exposed mice present increased IgG2a, IgE and IgM secretion. Groups of wildtype BALB/c mice were either subcutaneously infected with larvae (L3, L4), i.v. injected with MF or implanted with L5 or adult worms. On day 42 pi, lymphocytes isolated from infected BALB/c mice were left unstimulated or cultured with 100 µg/ml of the *Loa loa* developmental stage antigen extract that was originally used to infect the mouse. Antigen stimulation in cell cultures of lymphocytes from naive BALB/c mice served as control. Re-stimulation assays were cultured for 72 h at 37 °C and 5% CO_2_ and resulting levels of IgA (**a**), IgG1 (**b**), IgG2b (**c**), IgG3 (**d**), IgG2a (**e**), IgE (**f**) and IgM (**g**) were determined using the Luminex technology. Data show mean fluorescence intensity (MFI) of the different immunoglobulins from re-stimulated splenocytes of infected (closed symbols, *n* = 6 per life stage/parasite antigen extract) and naive BALB/c mice (open symbols, *n* = 3 per parasite antigen extract). Immunoglobulin levels were normalized by subtracting background levels of the comparable un-stimulated controls. Graphs show scatter plots with mean ± SEM. Statistically significant differences between the indicated groups were detected using the Friedman test followed by a Dunn’s multiple comparison test

Next, we analysed stage-specific recall in splenocyte cultures from groups of BALB/c mice that had been exposed to different *L. loa* life stages. In short, splenocytes were isolated on day 42 pi and re-stimulated with the antigen extract from the parasitic stage that was originally used to infect the individual BALB/c mouse. After 72 h of re-stimulation, released immunoglobulin levels in the culture supernatants of spleen cells from naive and exposed BALB/c groups were determined using Luminex technology (bead-based multiplex assay) and levels were normalized by subtracting background levels from matched cultures without antigen. In contrast to the systemic responses, significant differences were detected between the different groups (Fig. 1). Whereas there were no significant differences in levels of IgA, IgG1, IgG2b and IgG3 (Fig. 1a–d) between the different infection scenarios, re-stimulation with MF lead to significantly enhanced IgG2a (Friedman test: *F* = 10.63, *P* = 0.0311; Dunn’s *post-hoc* test: *P* = 0.0466), IgE (Friedman test: *F* = 14.68, *P* = 0.0054; Dunn’s *post-hoc* test: *P* = 0.0026) and IgM (Friedman test: *F* = 19.73, *P* = 0.0006; Dunn’s *post-hoc* test: *P* = 0.0140 and *P* = 0.0009) in MF-exposed mice when compared to cultures from groups exposed to L4 and adult worms and responses to their specific antigens (Fig. 1e–g). Interestingly, no immunoglobulins were detected in cultures from L4-exposed mice when stimulated with L4 antigen extract. On an individual basis, spleen cultures from the *L. loa*-exposed groups showed enhanced levels of immunoglobulins upon specific antigen stimulation compared to matched cell cultures from naive mice (open symbols). For example, cultures from mice given adult worms produced elevated IgA and IgE levels (Fig. 1a, f). Moreover, there was strong IgG2b release from cultures stemming from L3- and MF-exposed mice (Fig. 1c). Indeed, apart from IgE, cultures from MF-exposed mice produced high levels of all Ig subtypes. These initial findings suggest that MF exposure induces a defined Ig profile that can be recalled when MF have been eliminated.

### Reduced secretion of pro-inflammatory and Th1 cytokines in L3 and L4 antigen extract re-stimulated splenocytes

**Fig. 2:**
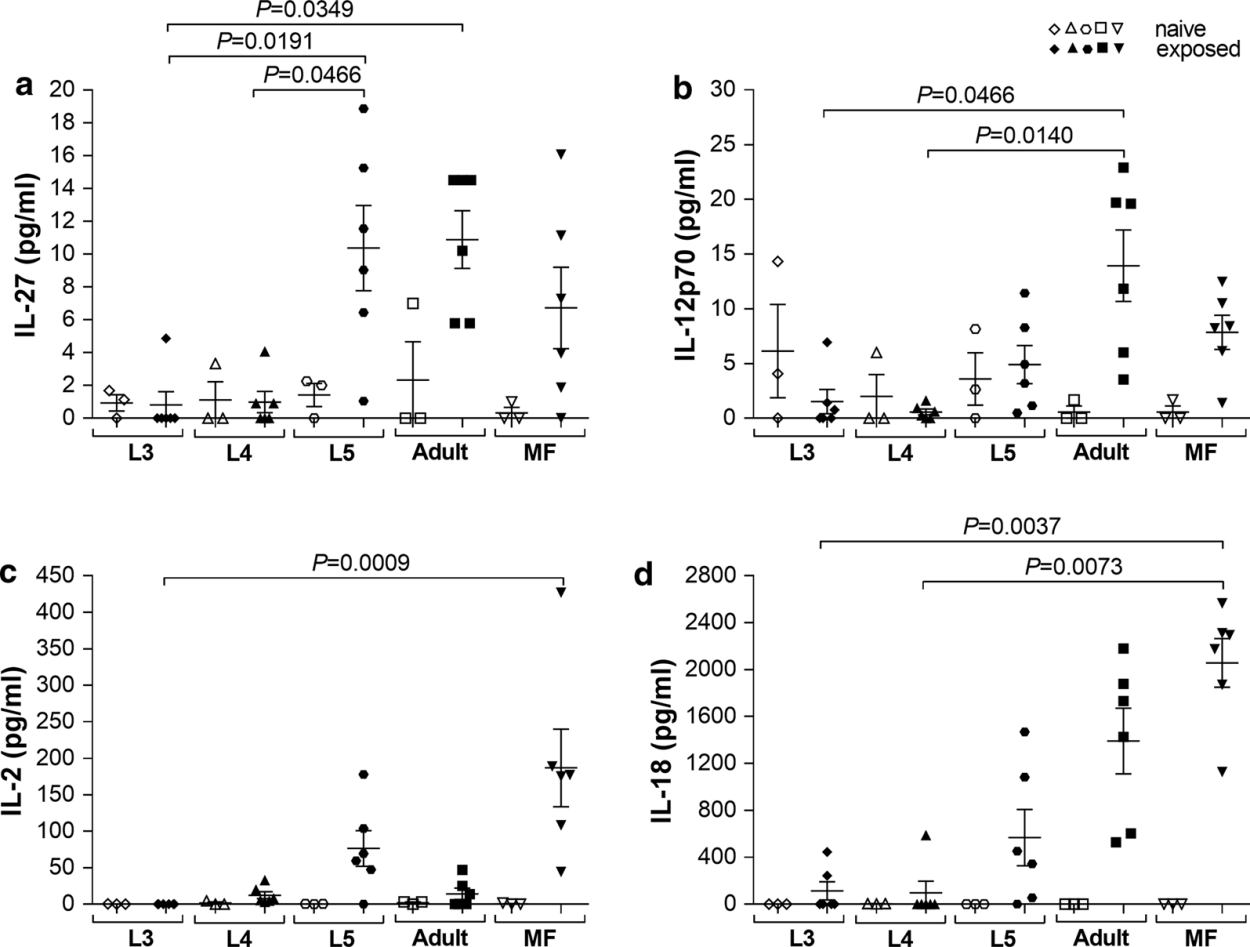
Reduced pro-inflammatory and Th1 cytokine responses in L3 and L4 antigen extract re-stimulated splenocytes. Groups of wildtype BALB/c mice were either subcutaneously infected with larvae (L3, L4), i.v. injected with MF or implanted with L5 or adult worms. On day 42 pi, lymphocytes isolated from infected BALB/c mice were left unstimulated or cultured with 100 µg/ml of the *Loa loa* developmental stage antigen extract that was originally used to infect the mouse. Antigen stimulation in cell cultures of lymphocytes from naive BALB/c mice served as control. Re-stimulation assays were cultured for 48 h at 37 °C and 5% CO_2_ and resulting levels of IL-27 (**a**), IL-12p70 (**b**), IL-2 (**c**) and IL-18 (**d**) were determined using the Luminex technology. Data show concentration (pg/ml) of the different cytokines from re-stimulated splenocytes of infected BALB/c mice (closed symbols, *n* = 6 per life stage/parasite antigen extract) and naive BALB/c mice (open symbols, *n* = 3 per parasite antigen extract). Cytokine levels were normalized by subtracting background levels of the comparable un-stimulated controls. Graphs show scatter plots with mean ± SEM. Statistically significant differences between the indicated groups were detected using the Friedman test followed by a Dunn’s multiple comparison test

In addition to immunoglobulin levels, we also analysed cytokine release to parasitic extracts in cultures of splenocytes from *L. loa*-exposed BALB/c mice after 48 h. Interestingly, whereas secretion of IFN-γ, TNF-α, IL-1β and IL-23 were not significantly different between the groups there were differences between recall responses of naive and stage-specific cultures (Additional file 6: Figure S6). For example, MF elicited both IFN-γ and TNF-α (Additional file 7: Figure S7). This was also reflected in release of IL-27 and IL-18 (Fig. 2). Interestingly, L5 and adult stages also released higher amounts of IL-27 and IL-18 when compared to responses in cell cultures from naive mice (Fig. 2a, d). Moreover, we also observed that re-stimulation with L5 and adult worm antigen extracts lead to higher IL-18 (Fig. 2d) responses and significantly increased secretion of IL-27 (Friedman test: *F* = 17.41, *P* = 0.0016; Dunn’s *post-hoc* test: *P* = 0.0191, *P* = 0.0349 and *P* = 0.0466) (Fig. 2a) and IL-12p70 (Friedman test: *F* = 14.89, *P* = 0.0049; Dunn’s *post-hoc* test: *P* = 0.0466 and *P* = 0.0140) (Fig. 2b) when compared to recall responses by splenocyte cultures from L3- and L4-exposed BALB/c mice to their respective antigens. In addition, MF re-stimulation resulted in significantly enhanced IL-2 (Friedman test: *F* = 18.36, *P* = 0.0010; Dunn’s *post-hoc* test: *P* = 0.0009) (Fig. 2c) and IL-18 (Friedman test: *F* = 20.28, *P* = 0.0004; Dunn’s *post-hoc* test: *P* = 0.0037 and *P* = 0.0073) (Fig. 2d) secretion compared to L3 or L3 and L4 stage re-stimulation, respectively. Although the direct comparison of cytokine levels from the *L. loa* antigen extract re-stimulated splenocyte cultures is difficult due to limited sample size, these initial findings suggest that L3 and L4 stage larvae did not overtly provoke pro-inflammatory and/or Th1 immune responses, whereas infections with L5, adult worms and MF elicited recall immune responses.

### Exposure to *Loa loa* adult worm antigen extracts leads to enhanced Th2 and Th17 recall responses to this life stage

**Fig. 3:**
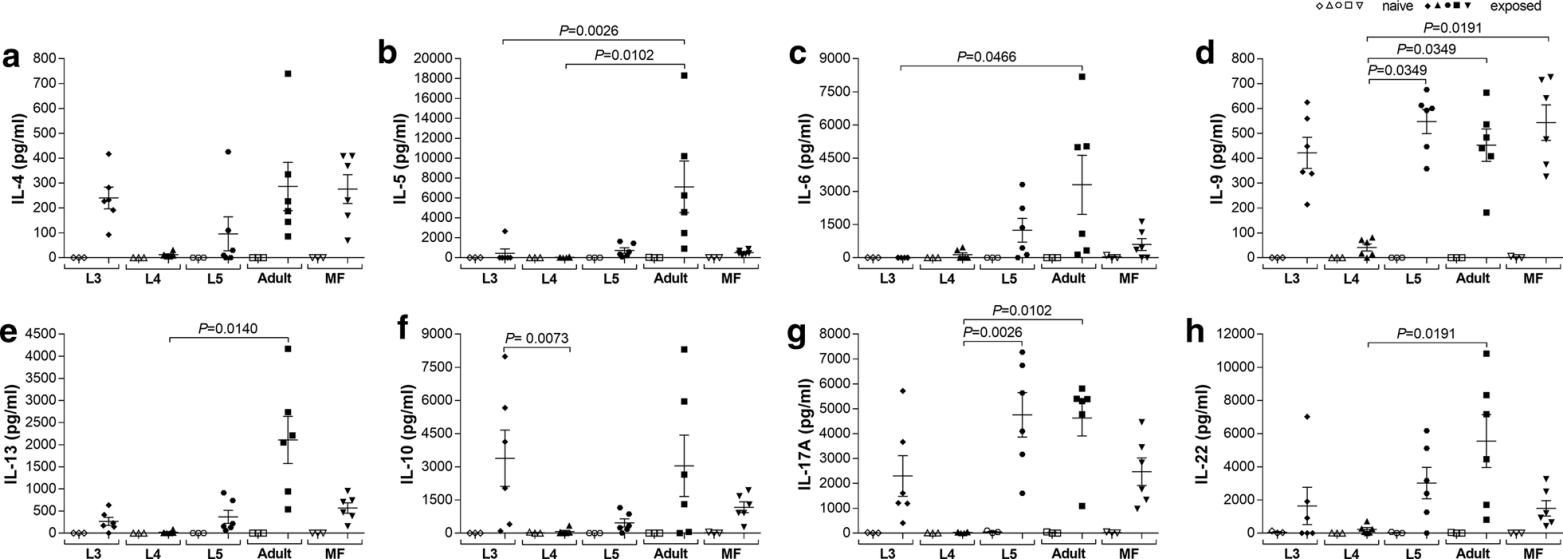
Increased Th2 and Th17 immune responses in adult worm antigen extract re-stimulated splenocytes. Groups of wildtype BALB/c mice were either subcutaneously infected with larvae (L3, L4), i.v. injected with MF or were implanted with L5 or adult worms. On day 42 pi, lymphocytes isolated from infected BALB/c mice were left unstimulated or cultured with 100 µg/ml of the *Loa loa* developmental stage antigen extract that was originally used to infect the mouse. Antigen stimulation in cell cultures of lymphocytes from naive BALB/c mice served as control. Re-stimulation assays were cultured for 48 h at 37 °C and 5% CO_2_ and resulting levels of IL-4 (**a**), IL-5 (**b**), IL-6 (**c**), IL-9 (**d**), IL-13 (**e**), IL-10 (**f**), IL-17A (**g**) and IL-22 (**h**) were determined using the Luminex technology. Data show concentration (pg/ml) of the different cytokines from re-stimulated splenocytes of infected BALB/c mice (closed symbols, *n* = 6 per life stage/parasite antigen extract) and naive BALB/c mice (open symbols, *n* = 3 per parasite antigen extract). Cytokine levels were normalized by subtracting background levels of the comparable un-stimulated controls. Graphs show scatter plots with mean ± SEM. Statistically significant differences between the indicated groups were detected using the Friedman test followed by a Dunn’s multiple comparison test

Besides pro-inflammatory and Th1 secretion patterns, we analysed other T cell-based immune responses and noted that whereas cell cultures from naive or L4-exposed BALB/c groups failed to produce any of the eight measured cytokines, comparable cultures from other *L. loa-*exposed mice produced significant cytokine responses to their respective antigens (Fig. 3). Interestingly, only co-cultures of cells from mice exposed to adult worms released IL-5 (Fig. 3b) and IL-13 (Fig. 3e). This single instance shows an element of specific cell priming during exposure since overlapping structures, epitopes and molecules between adult- and L5-exposed animals should have also perhaps provoked IL-5 release in L5-specific co-cultures.

When comparing the groups, re-stimulated splenocytes with adult worm antigen extract secreted significantly more IL-5 (Friedman test: *F* = 1 7.6, *P* = 0.0015; Dunn’s *post-hoc* test: *P* = 0.0026 and *P* = 0.0102) (Fig. 3b), IL-6 (Friedman test: *F* = 12.37, *P* = 0.0148; Dunn’s *post-hoc* test: *P* = 0.0466) (Fig. 3c), IL-9 (Friedman test: *F* = 13.47, *P* = 0.0092; Dunn’s *post-hoc* test: *P* = 0.0349) (Fig. 3d) and IL-13 (Friedman test: *F* = 18.27, *P* = 0.0011; Dunn’s *post-hoc* test: *P* = 0.0140) (Fig. 3e) compared to L3- or L4-stimulated cells, respectively. In addition, L5 and MF antigen extract re-stimulation only led to significantly increased IL-9 secretion (Dunn’s *post-hoc* test: *P* = 0.0349 and *P* = 0.0191) (Fig. 3d). Interestingly, only cultures from L3-exposed mice and antigen extract significantly increased the release of IL-10 secretion (Friedman test: *F* = 13.34, *P* = 0.0097; Dunn’s *post-hoc* test: *P* = 0.0073) compared to L4 stage stimulation (Fig. 3f). Since L3 transition into L4, it will be interesting to determine in the future whether the absence of L4-specific responses is associated with the lack of proceeding L3-provoked responses. In regard to Th17 responses, re-stimulation with L5 and adult worm antigen extract also significantly increased the secretion of IL-17A (Friedman test: *F* = 16.53, *P* = 0.0024; Dunn’s *post-hoc* test: *P* = 0.0102 and *P* = 0.0026) (Fig. 3g) and IL-22 (Friedman test: *F* = 11.93, *P* = 0.0149; Dunn’s *post-hoc* test: *P* = 0.0191) (Fig. 3h) compared to the L4 stage group. In summary, as shown with the pro-inflammatory and Th1 immune responses, L5, MF and especially adult worms triggered Th2 and Th17 responses whereas L4 stage larvae did not induce remarkable Th immune responses.

### Re-stimulation with antigen extracts from adult worms and MF induces distinct chemokine responses

**Fig. 4:**
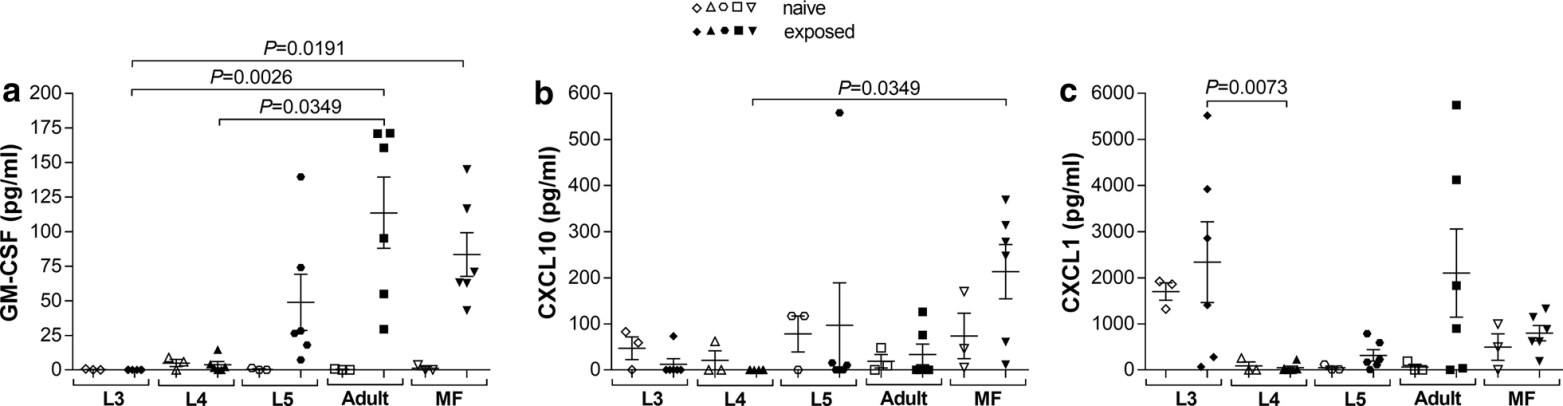
Adult worm and MF antigen extract induce distinct chemokines. Groups of wildtype BALB/c mice were either subcutaneously infected with larvae (L3, L4), i.v. injected with MF or were implanted with L5 or adult worms. On day 42 pi, lymphocytes isolated from infected BALB/c mice were left unstimulated or cultured with 100 µg/ml of the *Loa loa* developmental stage antigen extract that was originally used to infect the mouse. Antigen stimulation in cell cultures of lymphocytes from naive BALB/c mice served as control. Re-stimulation assays were cultured for 48 h at 37 °C and 5% CO_2_ and resulting levels of GM-CSF (**a**), CXCL10 (**b**) and CXCL1 (**c**) were determined using the Luminex technology. Data show concentration (pg/ml) of the different chemokines from re-stimulated splenocytes of infected BALB/c mice (closed symbols, *n* = 6 per life stage/parasite antigen extract) and naive BALB/c mice (open symbols, *n* = 3 per parasite antigen extract). Chemokine levels were normalized by subtracting background levels of the comparable un-stimulated controls. Graphs show scatter plots with mean ± SEM. Statistically significant differences between the indicated groups were detected using the Friedman test followed by a Dunn’s multiple comparison test

Finally, we determined chemokine recall responses upon stage specific re-stimulation of splenocytes from *L. loa*-exposed BALB/c mice. Whereas, secretion of MIP-1α, MIP-1β, MCP-1, MCP-3, MIP-2, eotaxin and RANTES were comparable between the groups (Additional file 7: Figure S7), adult worm and MF antigen extracts triggered higher secretions of GM-CSF, CXCL1 and CXCL10 (Fig. 4). In detail, adult worm antigen extracts significantly induced GM-CSF secretion (Friedman test: *F* = 20.07, *P* = 0.0005; Dunn’s *post-hoc* test: *P* = 0.0026 and *P* = 0.0349) from adult worm-exposed lymphocytes when compared to cultures from L3- and L4-infected animals with their specific life stage (Fig. 4a). In association, cells from MF-exposed mice produced more GM-CSF (Dunn’s *post-hoc* test: *P* = 0.0191) (Fig. 4a) and CXCL10 (Friedman test: *F* = 14.18, *P* = 0.0067; Dunn’s *post-hoc* test: *P*=0.0349) (Fig. 4b) when compared to cultures from L3- and L4-exposed mice and respective antigens. Interestingly, similar to IL-10 secretion (Fig. 3f), re-stimulation with L3 antigen extract led to significant production of CXCL1 (Friedman test: *F* = 13.34, *P* = 0.0097; Dunn’s *post-hoc* test: *P*=0.0073) (Fig. 4c). Overall, these findings from the initial set of experiments show that upon clearance of infection, *L. loa* has provoked long-lasting host memory that can elicit distinct secretion patterns of cytokines, chemokines and immunoglobulins. These *L. loa*-specific responses might contribute to immunity and protection against reinfection or shape immune responses to other infections.

## Discussion

Although *L. loa* infects *Mandrillus leucophaeus* and can be maintained in splenectomised baboons [25, 26], research about *L. loa*-induced immunological mechanisms have been understudied and mainly concentrated on the analysis of unspecific antibody responses [15, 27, 28]. A major reason for the limited understanding of loiasis-associated immune responses and clinical picture [29, 30] is the lack of appropriate infection models. Besides the established rodent models of *Brugia malayi* and *Onchocerca volvulus* [23, 31–33], we recently showed that BALB/c with impaired IL-4, IL-5 and IL-13 signalling as well as a lymphopenic γc-deficient strain allow development of *L. loa* life stages [22, 34]. This was also reflected in our studies with the rodent *Litomosoides sigmodontis* model since in IL-4Rα/IL-5^−/−^ BALB/c mice, worm burden and MF counts were significantly higher than in wildtype BALB/c control groups [35], suggesting that principally mice lacking Th2 responses provide a better environment for worm growth. However, these preclinical infection models have shown that host-adaptive immunity plays a crucial role for *L. loa* survival and fertility. Thus, to begin deciphering host immunity against *L. loa*, we analysed immune responses in immune-competent BALB/c mice upon exposure to different *L. loa* developmental stages. Previously, it was shown that *L. loa* cannot be maintained in wildtype BALB/c mice probably due to type-2 associated immunity [23, 36] that clears *L. loa* larvae after 1 week [24]. Indeed, we did not isolate any *L. loa* developmental stages 42 days pi which was reflected in the relatively comparable levels of systemic cytokine, chemokine and immunoglobulins between the infected groups. In accordance with these findings, we recently showed that systemic regulatory immune cell frequencies (regulatory B cells) had returned to basal levels in humans who cleared *Wuchereria bancrofti* infection due to anti-filarial treatment compared to uninfected endemic normals [37]. In association, some results did indicate a change in B cell activity since IgM and IgG levels in groups exposed to adult worms were lower than other groups which was accompanied by higher IL-27 (enhances germinal center B cell activity) [38] and IL-9 (IL-9R signalling in memory B cells regulates humoral recall responses) [39]. Nevertheless, these findings confirm and support that *L. loa* infections cannot be established in immune-competent BALB/c mice [24]. Interestingly, levels of permissiveness can be observed with other filarial, including the rodent-specific model *L. sigmodontis*. In that pre-clinical setting, mice infect all strains but are cleared in C57BL/6 mice after 40 days pi and in BALB/c mice, only a portion of infected mice become patent (MF release). The fact that adult worms can be produced in lymphopenic γc-deficient mice provides a much-needed platform for (i) obtaining the different life-stages to prepare antigen extracts; and (ii) elucidating cellular components involved in establishing infections *per se*. This model is equivalent to the RAG2IL-2Rγ^−/−^ C57BL/6 mice that we have shown to allow fully infections of *L. sigmodontis* and moreover, these mice present higher worm burden and MF counts [40]. All these upcoming models provide ways to show whether host immunity can establish filarial-specific memory to induce immune response upon *L. loa* reinfection or exposure to antigen leading to resistance or enhanced immune responses, respectively.

Interestingly, re-stimulation of splenocytes from MF-exposed BALB/c mice with MF antigen extract led to enhanced IgG2a, IgE and IgM secretion. Besides the association of IgE and active *L. loa* infection [12, 13, 15, 16], several studies about schistosomiasis revealed that antigen-specific IgE responses were associated with the duration of exposure [41] and resistance to reinfection [42–44]. Whereas re-stimulation with L4 antigen extract did not lead to significantly increased immunoglobulin, cytokine and chemokine secretion, antigen extracts from L5, adult worms and MF potently induced immune responses from splenocytes isolated from BALB/c mice that had cleared *L. loa* developmental stages, confirming previous studies suggesting an important role of Th1 and Th2 immune responses during *L. loa* infection [9, 14]. Since L5, adult worms and MF share an overwhelming majority of antigens also due to intrauterine MF in female worms, explains the overlapping induction of immune responses between these life stages. However, especially, adult worms induce distinct chemokines and especially Th2 and Th17 cytokines like IL-5, IL-9, IL-13 and IL-17A, which have been shown to play a crucial role in the host defence against filariae [17–19, 35, 45–49]. In addition, it has been shown that *L. loa* worm antigen extract strongly induced T cell proliferative responses in PBMC from individuals in villages with low *L. loa* transmission rates compared to PBMC from individuals in high transmission villages [50]. These data suggest that distinct developmental stages modulate immune responses and contribute to memory recall responses that influence immunity against *L. loa*.

The reason for the insufficient immune responses of the L4 group might be due to the different amounts of worm material in terms of lower antigen load of the L4 larvae compared to the other stages, especially L5, adult worms and MF. In addition, L3 larvae might be coated in fly-host molecules and thus were seen as “non-self” leading to stronger immune responses compared to the L4 larvae. However, a general limitation of immunological studies is to equalise the parasite load and amount of antigen *per se*. For example, parasite load in humans vary strongly (e.g. microfilarial load) [17–19, 37, 47, 49] and results from the filarial mouse model *L. sigmodontis* are also based on different parasite loads when the natural infectious route *via* ticks is used [35, 40, 48]. Thus, comparisons of immune profiles in the mice groups following exposure to the different life stages are difficult to interpret and need cautious conclusions. Thus, further studies need to elucidate if *L. loa* L4 larvae can induce immune responses in the proposed mouse model.

Interestingly, re-stimulation of L3-exposed splenocytes with L3 antigen extract significantly induced IL-10 and CXCL1 secretion. However, IL-10 secretion was also induced in splenocytes upon L5, adult worm and MF antigen extract by tendency, confirming that chronic filarial infections are characterized by a dominate regulatory environment involving the secretion of IL-10 [17–19, 37]. In addition, several studies have shown that neutrophil activation play an important role for protective immunity against L3 [51–55], suggesting that the neutrophil-specific chemokine CXCL1 might be involved as well. Since *L. loa* developmental stages were difficult to obtain from mice or human volunteers, the resulting mouse infection studies performed here were restricted including the overall number of *L. loa-*exposed mice in each challenge group, recall responses in both infected and naive groups and measurements and thus comparisons of *L. loa*-specific immunoglobulin levels with total amounts. These initial data sets are also only based on one large comparative infection study but consider that these initial findings will become the precedent of such immune-modulatory research in loiasis. Furthermore, we could not perform cross-over stimulation studies like culturing splenocytes from one stage-specific exposed *L. loa* group with all the different *L. loa* developmental stage antigen extracts, nor were we able to analyse different time points upon parasite challenge to decipher how long the *L. loa* life stages can survive. Moreover, it was not possible to unravel if location of the parasite stage accompanied with antigen-drainage and immune cell migration influences splenocyte recall responses. Thus, further studies are required to optimize these initial *L. loa in vitro* cultures so that additional mouse infections with higher amounts of parasites and antigen extracts can be obtained. Such studies will evaluate in more detail the specificity, involvement and interactions of the studied immunoglobulins, chemokine and cytokines both systemically and at the site of infection.

## Conclusions

Overall, this pilot study shows that infections with different developmental stages of *L. loa* were cleared within 42 days pi in immunocompetent BALB/c mice. Although no statistically significant findings were revealed in systemic immune parameters, re-stimulation of spleen lymphocytes with antigen extracts from each *L. loa* developmental life stage led to distinct immunoglobulin, cytokine and chemokine secretion patterns. Therefore, this pilot study highlights that the host immune system establish memory during an ongoing *L. loa* infection, leading to enhanced *L. loa*-specific recall responses in mice that cleared the infection. This mechanism might contribute to immunity and protection against reinfection.

## Supplementary information

**Additional file 1: Figure S1.** Overview of the study design.

**Additional file 2: Figure S2.** Comparable systemic immunoglobulin levels. Data show mean fluorescence intensity (MFI) of naive (*n* = 10) and BALB/c mice exposed to L3 (*n* = 5), L4 (*n* = 5), L5 (*n* = 5), adult worms (*n* = 5) or MF (*n* = 5).

**Additional file 3: Figure S3.** Comparable systemic pro-inflammatory and Th1 cytokine levels. Data show concentration (pg/ml) of the different cytokines from groups of naive (*n* = 10) and BALB/c mice exposed to L3 (*n* = 5), L4 (*n* = 5), L5 (*n* = 5), adult worms (*n* = 5) or MF (*n* = 5).

**Additional file 4: Figure S4.** Comparable systemic regulatory, Th2 and Th17 cytokine levels. Data show concentration (pg/ml) of the different cytokines from groups of naive (*n* = 10) and BALB/c mice exposed to L3 (*n* = 5), L4 (*n* = 5), L5 (*n* = 5), adult worms (*n* = 5) or MF (*n* = 5).

**Additional file 5: Figure S5.** Comparable systemic chemokine levels. Data show concentration (pg/ml) of the different chemokines from groups of naive (*n* = 10) and BALB/c mice exposed to L3 (*n* = 5), L4 (*n* = 5), L5 (*n* = 5), adult worms (*n* = 5) or MF (*n* = 5).

**Additional file 6: Figure S6.** Comparable IFN-γ, TNF-α, IL-1β and IL-23 levels of antigen extract re-stimulated splenocytes. Data show concentration (pg/ml) of the different cytokines from re-stimulated splenocytes of infected BALB/c mice (*n* = 6 per life stage/parasite antigen extract) and naive BALB/c mice (*n* = 3 per parasite antigen extract).

**Additional file 7: Figure S7.** Comparable chemokine levels of antigen extract re-stimulated splenocytes. Data show concentration (pg/ml) of the different chemokines from re-stimulated splenocytes of infected BALB/c mice (*n* = 6 per life stage/parasite antigen extract) and naive BALB/c mice (*n* = 3 per parasite antigen extract).

## Data Availability

The data supporting the conclusions of this article are included within the article and its additional files. The raw datasets are available from the corresponding author upon reasonable request.

## Abbreviations

L3: Third-stage larvale
L4: Fourth-stage larvae
L5: Fifth-stage larvae
MF: Microfilariae
SE: Standard error
