# Supplementary figures and images for: Comparison of immune responses to Loa loa stage-specific antigen extracts in Loa loa-exposed BALB/c mice upon clearance of infection
Source: Parasit Vectors. 2020 Feb 7;13:51. doi: 10.1186/s13071-020-3921-x (PMC7006431; doi:10.1186/s13071-020-3921-x)

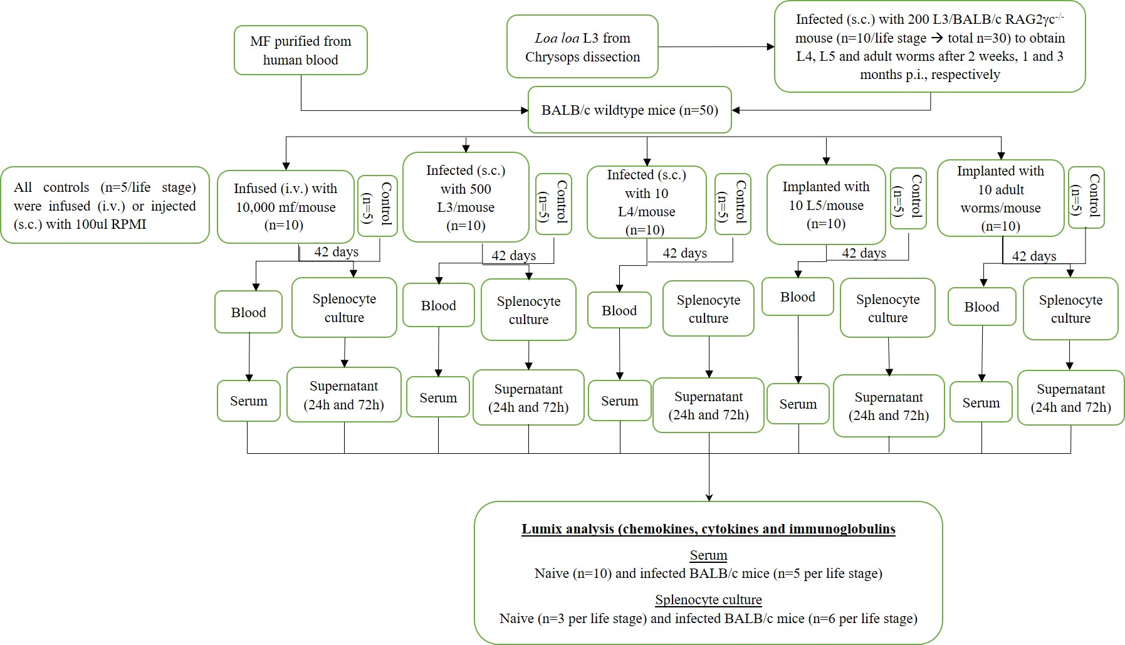

Supplement: Supplementary file 1 — Additional file 1: Figure S1. Overview of the study design. [file 13071_2020_3921_MOESM1_ESM.tif]

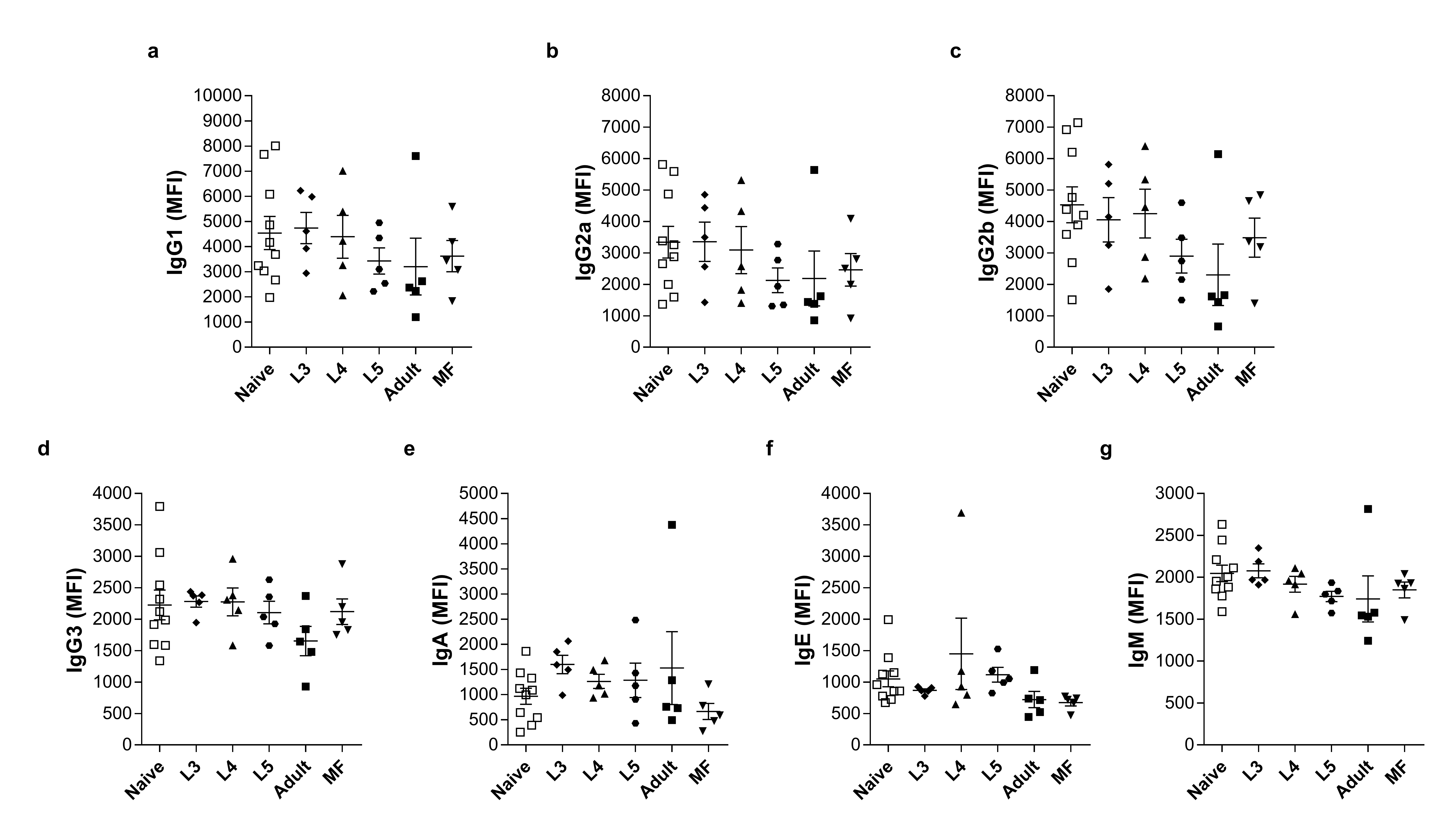

Supplement: Supplementary file 2 — Additional file 2: Figure S2. Comparable systemic immunoglobulin levels. Data show mean fluorescence intensity (MFI) of naive (n = 10) and BALB/c mice exposed to L3 (n = 5), L4 (n = 5), L5 (n = 5), adult worms (n = 5) or MF (n = 5). [file 13071_2020_3921_MOESM2_ESM.tif]

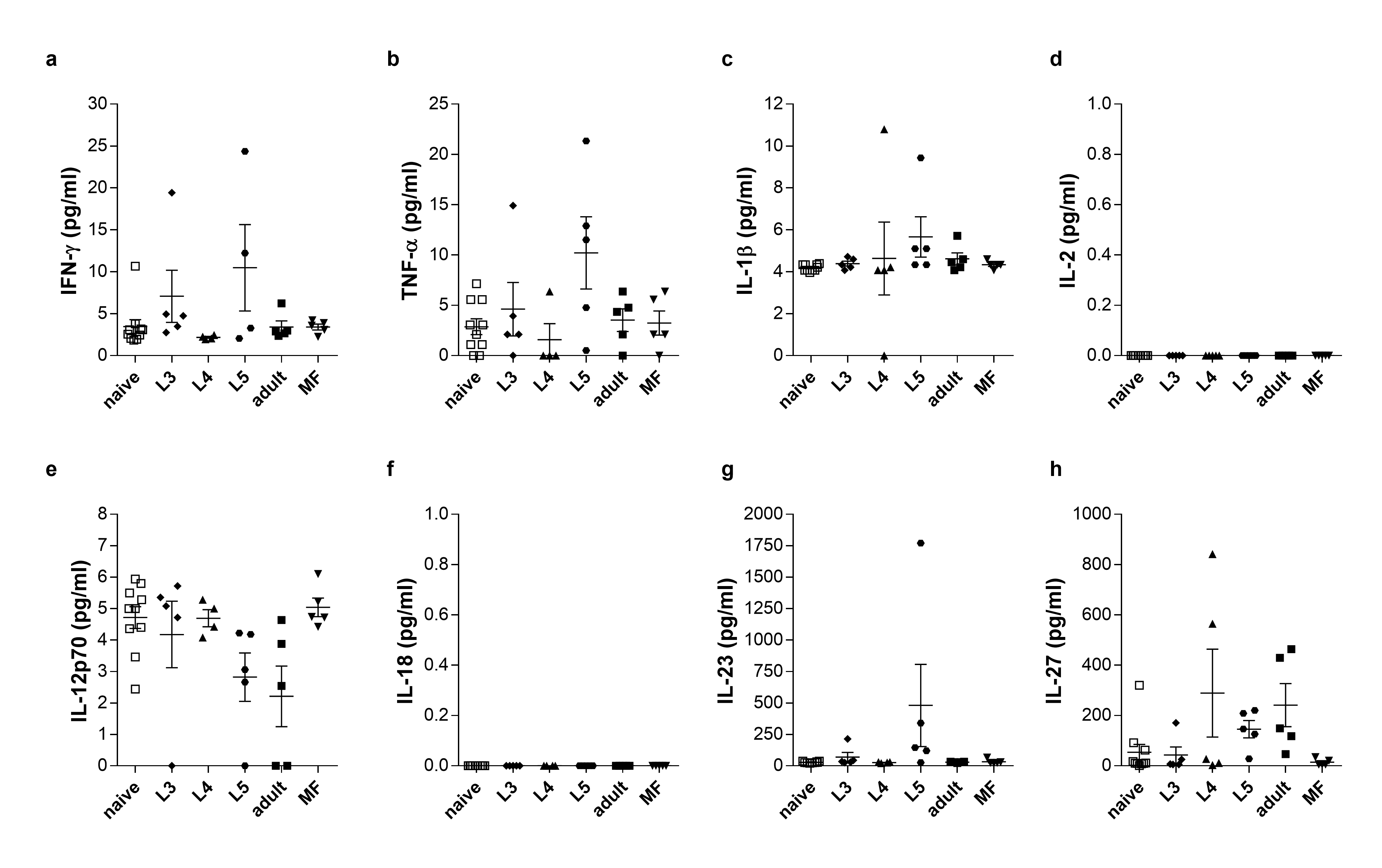

Supplement: Supplementary file 3 — Additional file 3: Figure S3. Comparable systemic pro-inflammatory and Th1 cytokine levels. Data show concentration (pg/ml) of the different cytokines from groups of naive (n = 10) and BALB/c mice exposed to L3 (n = 5), L4 (n = 5), L5 (n = 5), adult worms (n = 5) or MF (n = 5). [file 13071_2020_3921_MOESM3_ESM.tif]

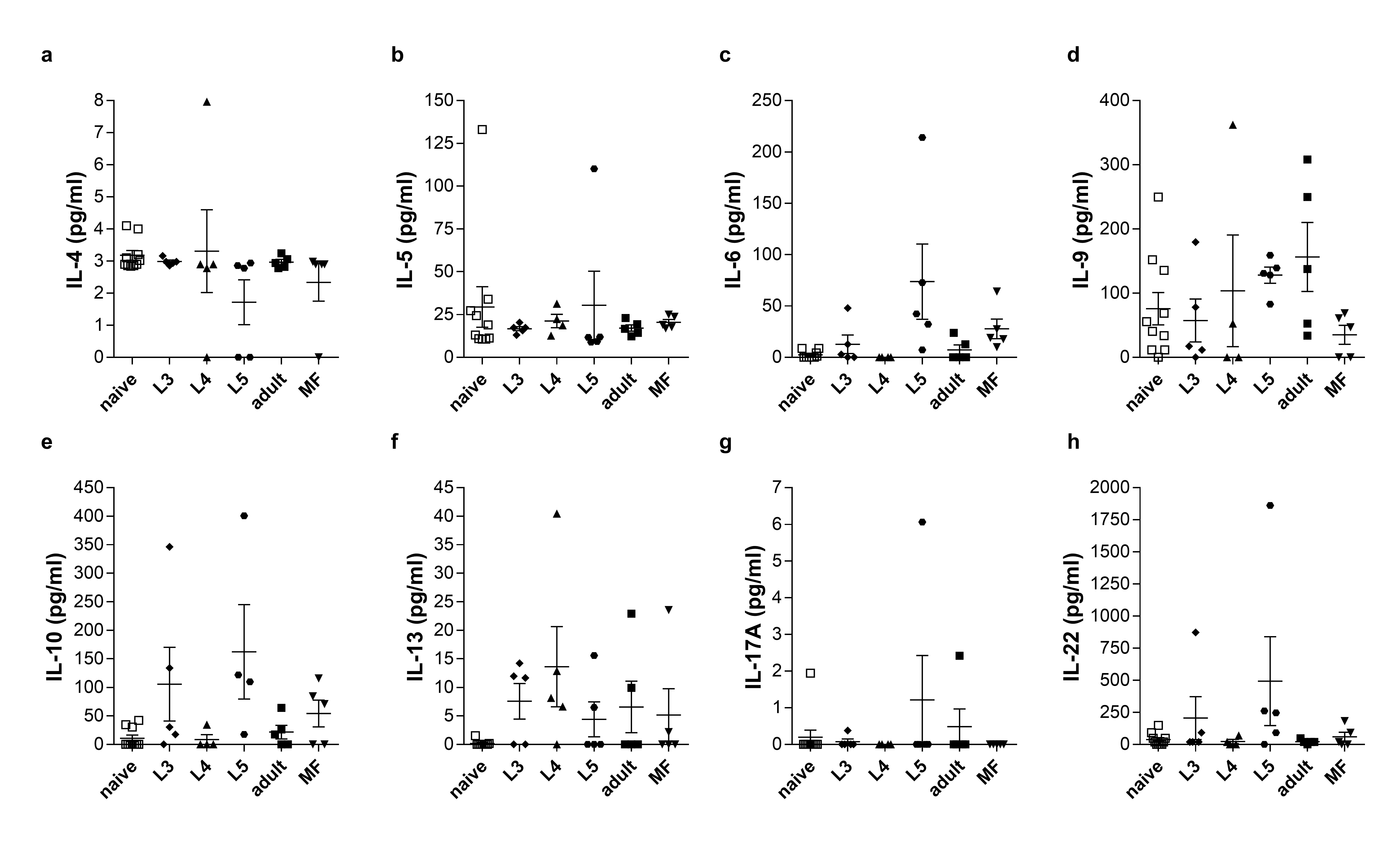

Supplement: Supplementary file 4 — Additional file 4: Figure S4. Comparable systemic regulatory, Th2 and Th17 cytokine levels. Data show concentration (pg/ml) of the different cytokines from groups of naive (n = 10) and BALB/c mice exposed to L3 (n = 5), L4 (n = 5), L5 (n = 5), adult worms (n = 5) or MF (n = 5). [file 13071_2020_3921_MOESM4_ESM.tif]

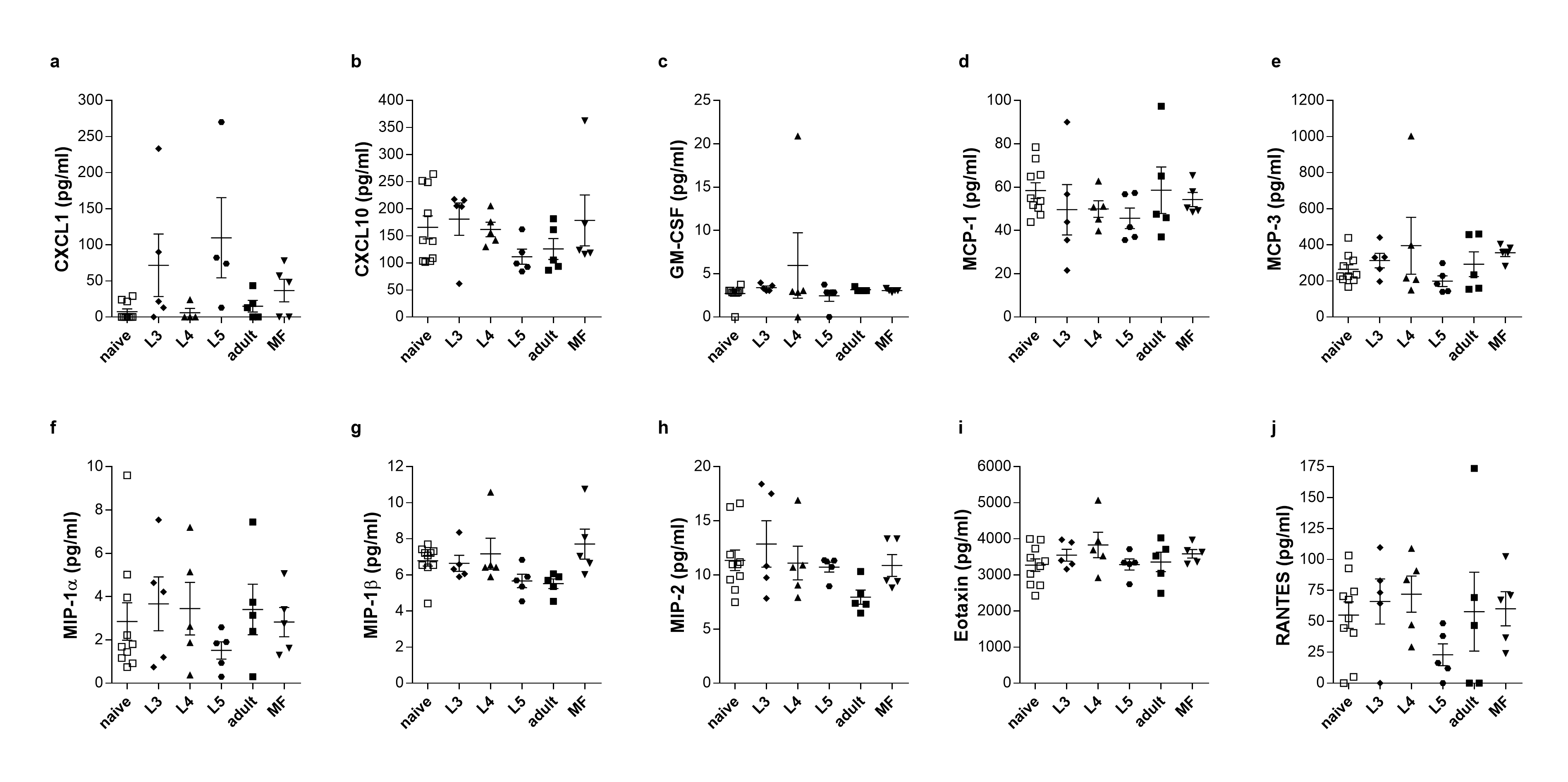

Supplement: Supplementary file 5 — Additional file 5: Figure S5. Comparable systemic chemokine levels. Data show concentration (pg/ml) of the different chemokines from groups of naive (n = 10) and BALB/c mice exposed to L3 (n = 5), L4 (n = 5), L5 (n = 5), adult worms (n = 5) or MF (n = 5). [file 13071_2020_3921_MOESM5_ESM.tif]

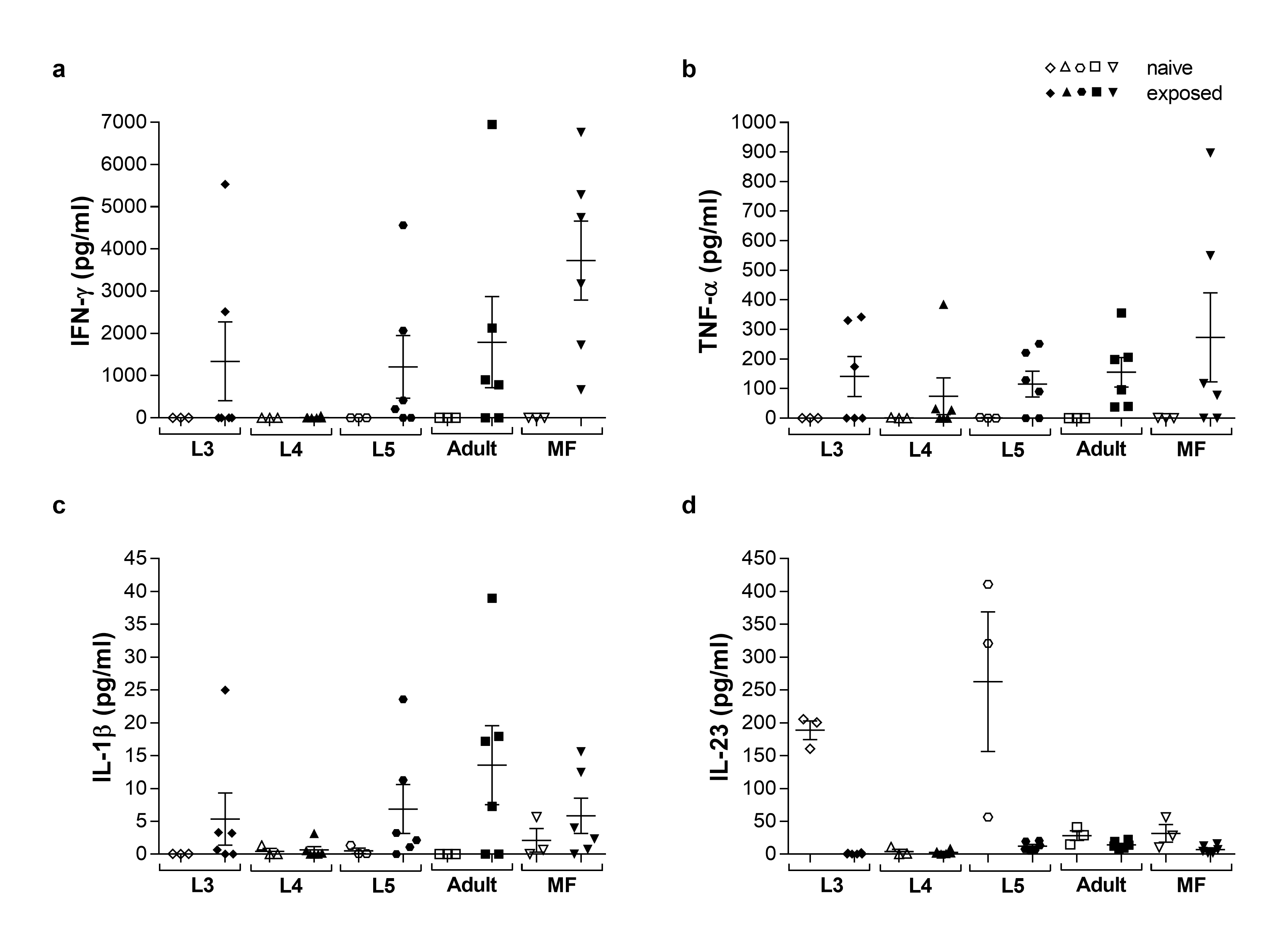

Supplement: Supplementary file 6 — Additional file 6: Figure S6. Comparable IFN-γ, TNF-α, IL-1β and IL-23 levels of antigen extract re-stimulated splenocytes. Data show concentration (pg/ml) of the different cytokines from re-stimulated splenocytes of infected BALB/c mice (n = 6 per life stage/parasite antigen extract) and naive BALB/c mice (n = 3 per parasite antigen extract). [file 13071_2020_3921_MOESM6_ESM.tif]

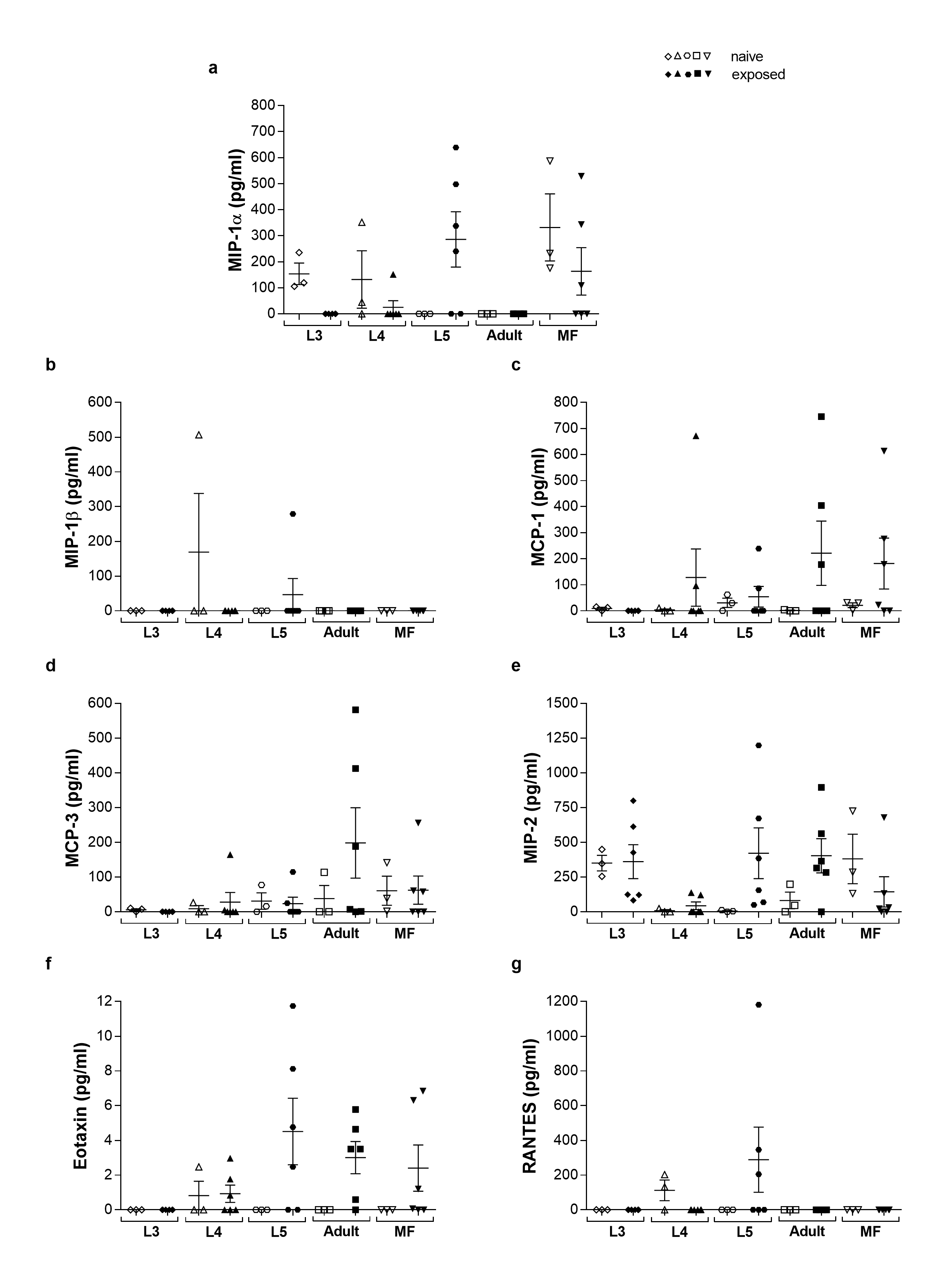

Supplement: Supplementary file 7 — Additional file 7: Figure S7. Comparable chemokine levels of antigen extract re-stimulated splenocytes. Data show concentration (pg/ml) of the different chemokines from re-stimulated splenocytes of infected BALB/c mice (n = 6 per life stage/parasite antigen extract) and naive BALB/c mice (n = 3 per parasite antigen extract). [file 13071_2020_3921_MOESM7_ESM.tif]
